# fast_protein_cluster: parallel and optimized clustering of large-scale protein modeling data

**DOI:** 10.1093/bioinformatics/btu098

**Published:** 2014-02-14

**Authors:** Ling-Hong Hung, Ram Samudrala

**Affiliations:** Department of Microbiology, University of Washington, Seattle, WA 98109, USA

## Abstract

**Motivation:** fast_protein_cluster is a fast, parallel and memory efficient package used to cluster 60 000 sets of protein models (with up to 550 000 models per set) generated by the Nutritious Rice for the World project.

**Results:** fast_protein_cluster is an optimized and extensible toolkit that supports Root Mean Square Deviation after optimal superposition (RMSD) and Template Modeling score (TM-score) as metrics. RMSD calculations using a laptop CPU are 60× faster than qcprot and 3× faster than current graphics processing unit (GPU) implementations. New GPU code further increases the speed of RMSD and TM-score calculations. fast_protein_cluster provides novel k-means and hierarchical clustering methods that are up to 250× and 2000× faster, respectively, than Clusco, and identify significantly more accurate models than Spicker and Clusco.

**Availability and implementation:** fast_protein_cluster is written in C++ using OpenMP for multi-threading support. Custom streaming Single Instruction Multiple Data (SIMD) extensions and advanced vector extension intrinsics code accelerate CPU calculations, and OpenCL kernels support AMD and Nvidia GPUs. fast_protein_cluster is available under the M.I.T. license. (http://software.compbio.washington.edu/fast_protein_cluster)

**Contact:** lhhung@compbio.washington.edu

**Supplementary information:** Supplementary data are available at *Bioinformatics* online.

## 1 INTRODUCTION

Many protein-structure prediction and protein-folding simulations generate a large ensemble of candidate structures using different starting conditions. By analyzing an ensemble of predicted structures, the overall consistency and accuracy of the final prediction can be increased. More accurate models are more structurally similar to the correct structure, and will thus, tend to be similar to each other and can be identified by clustering. Thus, finding models at the center of clusters is an effective method of identifying the best structures in an ensemble. The Nutritious Rice for the World project (http://protinfo.compbio.washington.edu/rice), an IBM World Community Grid project, generated *de novo* models of all modelable protein sequences in the rice proteome. More than 60 000 sets of protein models were generated with up to 550 000 models in a set. The size and the number sets exceeded the capability of existing clustering software.

fast_protein_cluster was written to analyze this large dataset using a Linux cluster consisting of 1200 CPU cores and five graphics processing units (GPUs). The new software is able to cluster a set of 450 000 protein models in 1.5 h on a single workstation node and clustered all 60 000 sets in six weeks. The fast implementation also makes it possible to use new clustering strategies, and we describe two methods that identify significantly higher quality models than the widely used Spicker (Zhang and Skolnick, 2004) and the recently published Clusco (Jamroz and Kolinski, 2013) packages.

## 2 IMPLEMENTATION

Clustering involves partitioning models into sets of similar structures. fast_protein_cluster implements k-means, and hierarchical clustering methods using RMSD or TM-score as similarity metrics. Both the calculation of structural similarity and the partitioning methods have been accelerated. Previously, we had described a new faster algorithm for RMSD and TM-score calculations (Hung and Samudrala, 2012). We now provide new multithreaded and SIMD assembler language implementations for RMSD and TM-score calculations for CPUs and a new faster GPU implementation for RMSD.

Hierarchical clustering is implemented using a new parallelized O(N^2^) algorithm (Müllner, 2013; Murtagh 2011: http://labs.genetics.ucla.edu/horvath/CoexpressionNetwork/Rpackages/flashClust) instead of the O(N^3^) hclust algorithm used in Clusco. k-means partitioning is implemented using a novel and much faster variant of the standard methodology. The faster and parallel approach is exploited in a multi-k-means strategy where multiple k-means partitioning solutions are generated from different random starting clusters and the best solution chosen using the criterion of maximum homogeneity. Finally, the memory usage is decreased using an optional compact 1 byte representation of the similarity matrix, which does not result in loss of clustering accuracy (see Supplementary Material). The main routines are written in optimized C++ using assembler intrinsics for SIMD code and OpenCL for GPU kernels. Much of the speed of the software, especially those of the partitioning methods, is because of algorithmic improvements that are independent of the hardware (see Supplementary Material). The code is portable and provides acceleration on hardware ranging from 10-year-old P4 CPUs to modern workstations and GPUs.

To assess the accuracy of the new multi-k-means and complete linkage hierarchical approaches, the models at the center of clusters generated by fast_protein_cluster were compared with the centroids from clusters generated by Spicker and Clusco. The test set consists of 56 ensembles of size 11 500–32 000 of models generated *de novo* by I-TASSER and used originally to test Spicker. Details of the algorithms, implementations, testing and additional benchmarks are provided in Supplementary Material.

## 3 RESULTS AND DISCUSSION

Existing clustering approaches have approximately the same performance on the Spicker set (Jamroz and Kolinski, 2013). However, Table 1 shows that the models at the centers of the clusters identified by fast_protein_cluster are superior to those identified by Spicker and Clusco. The improvements are statistically significant (*P* < 0.05) when comparing the best centroid model from the largest five clusters. To give some context to these differences, we note that the standard deviation of the average TM-score of model 1 from the top 20 groups at the recent critical assessment of protein structure prediction 10 was in the same range (0.016) (http://zhanglab.ccmb.med.umich.edu/casp10).

**Table 1.** Mean TM-score of centroids relative to native structure

Clustering method^a^	Centroid of largest cluster^b^	Best centroid of five largest clusters^b^
Spicker	0.584	0.607
Clusco/k-means/RMSD	0.585	0.612
Multi-k-means/RMSD	0.590	**0.624**
Multi-k-means/TM-score	0.592	**0.624**
Hierarchical/RMSD	0.588	**0.626**
Hierarchical/TM-score	**0.595**	**0.624**

**^a^**fast_protein_cluster k-means values are the average of five separate runs to control for different starting seeds. Distance matrices were calculated using CA-atom coordinates. **^b^**TM-score means that are significantly better (paired z-test with *P* < 0.05) than Spicker are in bold, and those significantly better than Clusco are underlined. The quality of the best model among the centroids of the five largest clusters is significantly improved when fast_protein_cluster is used as the clustering method.

The variability of *de novo* modeling can result in subpopulations of models that share different sets of locally correct structural features. These local similarities are detectable through their contribution to the global similarity metric. Complete-linkage hierarchical clustering uses the maximum distance between members in two clusters to determine which clusters to join. This is more conducive to the formation of more divergent final clusters of models that share common inconsistently predicted local features. Similarly, we attribute the improvement using multi-k-means method to its increased clustering accuracy resulting in better detection of the subtle effects of shared local similarity on the global metric.

**Fig. 1.**
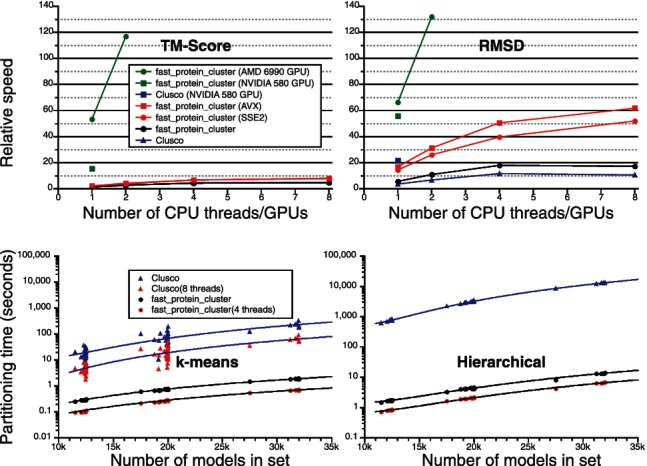
Performance of fast_protein_cluster. The speeds of all-atom RMSD and TM-score matrix calculations over the entire Spicker test set are shown relative to qcprot and the original TM-score for the different methodologies on a 4-core I7 CPU and two different GPUs. The times for k-means and hierarchical partitioning are shown as a function of the number of models. For RMSD calculations, the parallel SSE2 and AVX SIMD code on the laptop CPU outperform the Clusco GPU code. For partitioning, fast_protein_cluster is up to 250× and 2000× faster for k-means and hierarchical clustering, respectively

In Figure 1, we demonstrate significant improvements in performance on the Spicker test set. For TM-score calculations, the GPU approach has been previously described and we have extended support to Nvidia GPUs. The multithreaded SIMD-CPU implementation is new and is the fastest CPU version of TM-score, providing an increase of 80% over scalar code. For RMSD calculations, the new GPU implementation is several times faster than Clusco. The SIMD acceleration is especially effective for RMSD calculations, resulting in a 3–4-fold speedup over scalar code. On a single core, the CPU code achieves a 15-fold increase over qcprot (Theobald, 2005) and can match GPU speeds when using multiple threads. The increases in partitioning speed are even greater—up to 250-fold for k-means and 2000-fold for hierarchical clustering.

The speed and modular nature of fast_protein_cluster allow for exploration of new metrics and partitioning approaches on large sets of proteins. Its development has allowed us to cluster the data generated by the Nutritious Rice for the World project. Furthermore, clustering large sets is a common problem in bioinformatics. The algorithms and code are portable—user-defined similarity matrices are supported and new partitioning and input methods can be easily added to existing classes to extend the software’s functionality for applications beyond protein simulations. fast_protein_cluster is available under the permissive M.I.T. license. The source code, Makefile for Linux compilation, test set and documentation can be downloaded (http://software.compbio.washington.edu/fast_protein_cluster).
